# Supplementary material for: “We pray for the night to be shorter, so we can change our menstrual pads”: A qualitative exploration of menstrual hygiene challenges among internally displaced adolescent girls in Northern Ethiopia, 2023
Source: PLoS One. 2024 Oct 22;19(10):e0309985. doi: 10.1371/journal.pone.0309985 (PMC11495630; doi:10.1371/journal.pone.0309985)
Supplement: S1 Data — (DOC) [file pone.0309985.s001.doc]

# “We pray for the night to be shorter, so we can change our menstrual pads”: A qualitative exploration of menstrual hygiene challenges among internally displaced adolescent girls in Northern Ethiopia, 2023

# Project: MH among displaced students

Report created by Mobile46 on 6/7/2023

**Code Report**

Selected codes (28)

**○ Challenges for MH_ No antipain during menstruation**

**6 Quotations:**

**2:12 ¶ 41 in FGD2**

P3: now, it is a kind of so so (fifty-fifty )….emmm…..menstruation is difficult and when it comes it is so painful and there is no anti-pain.

**2:20 ¶ 45 in FGD2**

Besides, for example, me I feel strong pain and get sick during menstruation and I do not get medicine for that. While I was at my home before I came here, I used to get medicines to manage my pain during menstruation. But now I have no option and I do not get the medicine. So I have to suffer the pain and sleep for three consecutive days till the menstruation ends.

**4:5 ¶ 45 in IDI-2**

because many girls are suffering because of this. Many menstruating girls suffer from pain and are not getting medicine, menstrual pad and underwear.

**4:17 ¶ 56 in FGD 4**

P2: When I feel abdominal pain during menstruation, I go to the clinic but I don’t get medicine on time. So you simply waste your time. So everything is difficult in here. Because males and females are living together and you feel ashamed. You cannot wash your menstrual pad in front of males. So the problem in females is so huge.

**4:18 ¶ 57 in FGD 4**

P: In here we do not get anti-pain or any other medicine when we are sick. You might feel abdominal cramp or headache…..for example, I, myself suffer from headache and abdominal pain when my menstruation appears. So, I use to go to the clinic and got anti-pain. But now there is shortage of medicine and you do not get anti-pain

**4:19 ¶ 59 in FGD 4**

P6: There is no time that passes without pain during menstruation in almost every female. In this camp, the clinicians come only once in a week. So you do not get anti-pain if you have menstruation in the absence of the doctors.

**○ Challenges for MH_ Toilet too busy**

**1 Quotations:**

**4:12 ¶ 51 in IDI 3**

…Using the toilet is inconvenient as many people are using it, and if we decide to use it [toilet] we feel ashamed and are forced to leave almost immediately. This is because if we stay inside the toilet for a longer time, people will suspect that we are doing something (such as changing a menstrual pad) and they will try to see inside through the door hole…

**○ Challenges of using cloths for MH_ Lack of water**

**4 Quotations:**

**1:12 ¶ 45 in FGD1**

There is lack of soap and to some extent water

**2:5 ¶ 38 in IDI 3**

We use pieces of clothes and you will be ashamed of using that because you do not get water and soap,

**2:6 ¶ 38 in FGD2**

We use pieces of clothes and you will be ashamed of using that because you do not get water and soap, you have no many and you do not tell to your family to buy you menstrual pads.

**3:10 ¶ 65 in FGD 3**

Besides, there is shortage of water here and we are forced to go far to bring water.

**○ Challenges of using cloths for MH_ No privet place to dry it**

Comment: by Mobile46

The girls feel ashamed to hang their modes or torn cloths used for MH in public. They live in teh IDP enters aor in group and there is no privet place to dry them. In addition, teh washable modes demands longer time to dry than normal cloths, which affects the grils' decision to dry in the public.

**16 Quotations:**

**1:4 ¶ 44 in FGD1**

P2: In here, when we are using the washable modes, we are living in one room and we do not have place to dry it and many people can see it and you will be ashamed of it (tiskefi)….but when we were at our home, we had enough space to dry it and we were using the use and throw modes and that was good

**1:9 ¶ 44 in FGD1**

we may not even wash it because you do not have a space to dry it, or you might plan to wash it later and get spoiled…emm it is so bad.

**1:11 ¶ 45 in FGD1**

and it is difficult to wash it and dry it

**1:17 ¶ 47 in FGD1**

P6: drying normal clothes and modeses outside cannot be the same. People will be talking about it (modes). For me it is difficult to dry washable modes and underwear outside and how can you hang it inside because it will be spoiled and will have bad odour and it is meaningless.

**1:19 ¶ 49 in FGD1**

P7: Yes!we encounter such problems. Because we feel ashamed and we put it with out washing and drying it….and get spoiled. Then will not have other modes to use in the middle .

**1:20 ¶ 51 in IDI 6**

emmm….starting from my self..…I throw more modeses than the modese I wash. Because I don’t t wash the modeses as I can not dry them either inside or outside so I use to throw more modeses and I do not have use-and-throw modeses.

**1:21 ¶ 56 in FGD1**

P8:I, my self, throw many modeses than I use. Because, I do not get soap for washing, a place to dry it and you get worried…..and….many things…

**1:23 ¶ 59 – 60 in FGD1**

Probing: Do you get worried about as we worry about for food or anything else. For example we females have additional burden than males?

P6: yes, we worry as the menstruation period comes. You do not have the freedom and you got worried. We do not dry it outside with freedom like other clothes. As my colleagues mentioned, more modes are thrown away……

**1:27 ¶ 66 in FGD1**

Besides, the washable modes is difficult to dry. You will be in difficulty whether you wash it or not. You have no option and it is meaningless even if you have the washable modes.

**2:31 ¶ 57 in FGD2**

P4: it is embarrassing to hang the menstrual pads to dry. Particularly to dry them up in the sunny areas, many people can see it and you get embarrassed. Hence, you are obliged to put inside or hide it. Hence, you might reuse it while it is wet.

**2:33 ¶ 59 in FGD2**

P3: You have no freedom here. Even you have no freedom inside your home because while you are at home a guest might come and you cannot change the pad while the guest is in. Besides, it is also embarrassing to hide and take it outside…. for example to the toilet as the guest might think of that you are doing something different and unusual. You feel shy even in the presence of your father or elder brothers. Hence, you have to wait long till they leave the house;

**2:34 ¶ 59 in IDI 4**

we are living in one room and we can no go to the toilet while hiding it. Because, when you do like that they will be aware of that or may think differently…like what is she hiding from us? So we have to wait long till they leave.IN the mean time you suffer a lot.

**3:7 ¶ 62 in FGD 3**

P6: when you are living with many people, you feel ashamed to change, wash, and dry your menstrual pad. So you will be forced to wash your pad during the night so that others will not see you and then you try to dry it in a place where others cannot see it. But that is very difficult to find as there are many people around.

**3:8 ¶ 63 in FGD 3**

P7: as we are using pieces of clothes as menstrual pad, we try to wash the pad and dry it. But you have to dry it where people cannot see it, and we have to go to the toilet change the pad.

**4:11 ¶ 51 in FGD 4**

P6: we do not even have a place to dispose the used menstrual pads. We, sometimes, are forced to put the used menstrual pad in between our clothes. Because, we do not want it to be seen by others. We do not have even space to dry up washed clothes used for menstrual purpose.

**4:13 ¶ 51 in FGD 4**

So the only chance we have to wash our menstrual pad is during the night but the problem is how and where can you hang it to dry? So we have a problem still in drying the menstrual pad….

**○ Challenges of using cloths for MH_ No Soap**

**9 Quotations:**

**1:10 ¶ 45 in FGD1**

Menstruation is a blessing gifted for females. But now we are displaced from our homes and we are in difficulties. We do not got washing tools (like soap), before we were using the use-throw modes (Menstraul pads) but now we are not even getting the services including modes (Menstraul pads).There is lack of soap and to some extent water

**1:18 ¶ 47 in IDI-5**

Besides, it needs soap and omo (powder soap) and so many things……

**1:21 ¶ 56 in FGD1**

P8:I, my self, throw many modeses than I use. Because, I do not get soap for washing, a place to dry it and you get worried…..and….many things…

**1:22 ¶ 56 in FGD1**

and with the current situation it is very difficult to beg and there is shortage of money to buy normal or use-and-throw modes.

**2:2 ¶ 37 in FGD2**

To keep our hygiene….. we need soap and other things but we are not getting these things.

**2:6 ¶ 38 in FGD3**

We use pieces of clothes and you will be ashamed of using that because you do not get water and soap, you have no many and you do not tell to your family to buy you menstrual pads. We can’t find old cloths easily [to tear and use to make pads]. While we were at home, we used to find as many clothes as we needed. However, here [in the camp] we don’t even have enough clothes to change we cannot tear up clothes for that purpose [menstrual absorbent] as there is a critical shortage of clothes. We suffer a lot because of menstruation, and we always curse the day we were born

**2:9 ¶ 39 in IDI6**

While we were at our own home, we had full access to menstrual hygiene management tools. We used to get disposable menstrual pad and soap.

**2:10 ¶ 39 in FGD2**

But in here we are using the washable (re-usable) pad but we do not get soap to wash our menstrual pad and we do not tell our families to give us money or buy…..they do not afford it.

**2:13 ¶ 41 in FGD2**

we do not get modes and if we get the washable pad it is not comfortable and we face difficulty in reusing it as there is no soap to wash it. … and it is very difficult to tear our clothes for menstrual hygiene. If we plan to use our clothes as a menstrual pad, we will have a shortage of clothes and we cannot tear our clothes for this purpose, but we are using our pajamas or whatever we have.

**○ challenges of using cloths for MH_ Not comfortable**

**1 Quotations:**

**4:2 ¶ 44 in FGD 4**

We are not getting menstrual pads and when we are using clothe instead, it created friction and it is damaging our skin. It is not comfortable.

**○ Challenges of using cloths for MH_ remained stained**

**2 Quotations:**

**1:5 ¶ 43 in FGD1**

when we are using pieces of clothes, in some of the clothes the blood can be washed away easily, but in some other clothes the blood cannot be washed away and remains stained.

**1:8 ¶ 44 in IDI3**

But when we are using the washable (re-usable) modes, there are easily washable and some others are not easily washable

**○ Challenges of using cloths for MH_ Shortage of cloths**

**10 Quotations:**

**1:26 ¶ 65 – 66 in FGD1**

Probing: Do you get the pyjmammas or pieces of clothes easily to use for menstruation purpose?

P7: It is very difficult to tear your clothes for the purpose of menstrual hygine. If you plan to use your clothe as a modes, you will have shortage of clothes and you cannot tear your cloth for this purpose……but we are doing it from our pyjamas or from what we have because it is easy to dry and nobody will recognize that it is used for menstrual purpose.

**2:17 ¶ 42 in FGD2**

Therefore, we are forced to use pieces of clothes. But it is still too difficult to find soft clothes and we are waiting for support.

**2:18 ¶ 44 in FGD2**

P1:We do not even find the old clothes easily.While we were at home, we used to find clothes as needed but in here we have no extra clothes. We cannot tear clothes for that purpose as there is critical shortage of clothes. We suffer a lot because of menstruation and we always curse the day we were borne.

**2:19 ¶ 45 in FGD2**

P6: you know…..to find clothes for menstrual, we are tearing our normal clothes that we are wearing and we do not have extra cloth even for change. Hence, we are at very critical need of menstrual pads and we hope if any organization can give us.

**2:22 ¶ 46 in FGD2**

P7: In using old clothes for menstrual purpose, my friends have already explained it well. Everybody leaving here is almost naked, we have no clothes, and we do not find any cloth to tear for that purpose and I urge if any one can help us.

**2:29 ¶ 55 in IDI 2**

The only option you have is to use clothes but the cloth is not good enough to absorb the blood. You might tear many clothes but that is useless.

**2:30 ¶ 53 in FGD2**

P6: You do not have freedom to change your modes because you cannot change your modes as many of your family members are at home. You will be ashamed of that besides the clothes we are using are so thin and cannot absorb the blood well so the blood will stain your cloth.

**3:11 ¶ 67 in FGD 3**

P10: Because you might be frightened to go far and you will be forced to throw your pad as it not washed. So you will be faced to another problem, that is, shortage of pads and you have to tear another cloth for that purpose.

**3:12 ¶ 68 – 69 in FGD 3**

robing: How do you get the clothes for this purpose? Do you have the access to these clothes?

P10: No we have not. We are using what so ever cloth we find or we have to tear another cloth. We beg clothes from relatives. But you do not say it is for the purpose of pad but you make a reason that you want to wear it just you beg normal clothing. We girls do not ask clearly because it is embarrassing.

**3:13 ¶ 70 in FGD 3**

P4: As has been said, you will embarrassed to disclose. You might have some pieces of clothes but if you do not have, you will tear from your normal clothing or you beg from your friends.

**○ Challenges to MH_ Girls feel ashamed to communicate their family memebers about MH**

**9 Quotations:**

**1:39 ¶ 83 in FGD1**

P:It is so challenging, it is very difficult to change when it appears during meal time especially in front of your families and among your families there will be there will be elderly people, and you do not dare to search for modes or any piece of clothe for that purpose. What I do is I make a reason that I am sick of another sickness and try to escape from that. You might take water to the toilet but never modes while you families are watching you.

**1:40 ¶ 84 in FGD1**

P8: If it happens suddenly, you do not tell to anybody even to your mother or sister, you can simply have water and go to the toilet to wash. You do not have freedom at all and you do not expose yourself to anyone else. When the menstruation happens, we might feel back pain or abdominal pain but you do not tell to anybody this and that because you feel shy to talk about.

**2:8 ¶ 38 in FGD2**

you have no many and you do not tell to your family to buy you menstrual pads.

**2:10 ¶ 39 in IDI 4**

But in here we are using the washable (re-usable) pad but we do not get soap to wash our menstrual pad and we do not tell our families to give us money or buy…..they do not afford it.

**2:24 ¶ 48 in FGD2**

P1: I feel shy to change. But we have no option. But if we are with our mothers, we do not worry that much. But if we are with our fathers and elder brothers, we feel sh

**2:41 ¶ 67 in FGD2**

You do not tell to your family to accompany you because you will be embarrassed to tell them you are menstruating and going to change your pad.

**3:14 ¶ 72 in FGD 3**

P10: If your are living with your own family, you will inform to one of your sisters or may brothers to accompany you to go to the toilet to change your pad. But you cannot change the menstrual pad in the room because there people other than your family. If you do not have sister or brother, you will be forced to tell other people may be your peer to accompany you to the toilet. Because it is so scary to go alone to the toilet during the night.

**4:7 ¶ 48 in FGD 4**

P4:It is quite different while we were at home and in here in the management of our menstrual hygiene. This is the worst condition we are having. In here, when your menstruation comes, you do not get menstrual pad, you do not get soap, you do not get underwear. You need to have pads to change it with some interval (for example you have to change it after half a day), and you need soap to wash your pad. We are so much worried here….the worst things we have seen earlier are coming to our mind and disturbing us.

**4:10 ¶ 50 in IDI 1**

Though you wash your pads, you do not get space to dry it. So when the menstruation comes, we get ashamed of it because as we do not have sanitary materials and our clothes are soaked with blood and we are totally ignored and considered as below human. We are hurt very much.

**○ Challenges to MH_ No light in the toilet during night to chnage modes**

**2 Quotations:**

**1:31 ¶ 71 in FGD1**

P3: Let me share what happened (encountered) to me once upon a time. One night there was no light and I had to lit candle and go to the toile and sadly the candle was blown off by the wind (tefi’a) then I really suffered and was difficult to change the modes.

**3:11 ¶ 67 in FGD 3**

P10: Because you might be frightened to go far and you will be forced to throw your pad as it not washed. So you will be faced to another problem, that is, shortage of pads and you have to tear another cloth for that purpose.

**○ Challenges to MH_ No money to buy sanitaion pads**

Comment: by Mobile46

5/22/2023 4:37:23 PM, merged with Challenges for MH_ No mony to buy sanitation pad

**3 Quotations:**

**1:33 ¶ 74 in FGD1**

P8 Emm…when it [menstruation] happens, while we were at home, I mean…before we were displaced, we used modess [disposable pads] but now we cannot get modess and we are using pieces of clothing

**2:7 ¶ 38 in FGD2**

you have no many and you do not tell to your family to buy you menstrual pads.

**2:15 ¶ 42 in FGD2**

P2: Currently, I am not using menstrual pads. But when I was at home, I used to buy every type of menstrual pad. But now I have nothing [money] to buy with. I could not even ask my parents for money to buy pads Emm…not only menstrual pads, but they also [parents] don’t even have money to buy food.

**○ Challenges to MH_ No one accmpany us in the night to chnage modes**

**1 Quotations:**

**1:30 ¶ 70 in FGD1**

P7:Emmm….. It depends…it depends on your relationship. If you have your brother you might invite him to accompany you.

**○ Challenges to MH_ No Sanitation Pads**

**1 Quotations:**

**1:41 ¶ 86 in FGD1**

P1: we get it very rarely.

**○ Challenges to MH_ Privecy issue and Feel Ashamed**

**10 Quotations:**

**1:29 ¶ 68 in FGD1**

it is difficult to change your modes in front of your family even your brothers especially in the presence of guests. You do not dare it even if you are not shy. It is very difficult to do so in our society and is not acceptable.

**1:34 ¶ 74 in IDI3**

Oh, it is very difficult! When menstruation comes at night we cannot go to the toilet because it is so far away. We cannot change the pads inside our home because it is dark inside and if we try to search for a pad at home during the night everybody will be awakened and asked, ‘What is going on with you?’ So, we prefer to keep silent and wait until morning

**1:38 ¶ 82 in FGD1**

P: You cannot change that or hold modes with your hand while your families are around. I feel shy…... If I feel I am soaked, I automatically leave the house and move to the toilet to wash. Any ways it is difficult for me to search for modes in front of my family….they will see you when you seach. For me it is difficult. Besides, you might not have modes ready for that.

**1:39 ¶ 83 in FGD1**

P:It is so challenging, it is very difficult to change when it appears during meal time especially in front of your families and among your families there will be there will be elderly people, and you do not dare to search for modes or any piece of clothe for that purpose. What I do is I make a reason that I am sick of another sickness and try to escape from that. You might take water to the toilet but never modes while you families are watching you.

**1:40 ¶ 84 in FGD1**

P8: If it happens suddenly, you do not tell to anybody even to your mother or sister, you can simply have water and go to the toilet to wash. You do not have freedom at all and you do not expose yourself to anyone else. When the menstruation happens, we might feel back pain or abdominal pain but you do not tell to anybody this and that because you feel shy to talk about.

**2:23 ¶ 47 – 48 in FGD2**

Probing questions: As you mentioned, you do not have soaps, water and clothes for that purpose. What about regarding your freedom in changing menstrual pads while your families are inside as you are living in single rooms with your families?

P1: I feel shy to change. But we have no option. But if we are with our mothers, we do not worry that much. But if we are with our fathers and elder brothers, we feel shy. If the menstruation comes, I have no problem in telling about the situation to my mother and what I do is I go to the toilet and change.

**2:25 ¶ 49 – 50 in FGD2**

Probing: Have you ever/a friend or sister experienced menstruation on the way you were moving from your original home to Mekelle?

P2:Yes, one of my sisters has experienced that. She experienced menstruation while were moving. There were many people and she could do nothing and we had no clothes so she tore from her own pyjamma, and me and other girls covered her with the intention that others could not see her while she was changing. Therefore, we girls do not have freedom.

**2:26 ¶ 52 in FGD2**

P7: If menstruation happens suddenly while your family members are at home, you get ashamed and you cannot change your modes. Hence, you need to go to the toilet or bath room to change. But this more worse when the menstruation comes while you are outside your home or in front of unfamiliar people to you. Additionally w*e do not have the* freedom to do as we wish, so we get worried. We do not hang the pads outside freely as we do with other clothes. As my friends mentioned before, we throw away reusable pads and clothes, which could have been reused if we could have washed and dried them

**3:5 ¶ 60 in FGD 3**

P7: You do not have any other room to change your menstrual pad. What you can do is to be in one corner of the room and cover (hide) yourself with any cloth so that you try to change. The worst thing is while you change, there could be even more people rather than your family in the house. So that is so difficult.

**3:6 ¶ 61 in IDI 6**

P4**:** It is quite different from what we were doing at home [menstrual hygiene management]. This is the worst condition we have ever had in our life. When our menstruation comes, it is scary, especially when we know we do not have a menstrual pad! We do not get soap! We do not get underwear! We need pads to change at intervals and soap to wash our pads, but we have none! We are so worried here [in the camp!].

**○ Challenges to MH_ Toilet is remot_ risk of rape**

**10 Quotations:**

**1:32 ¶ 73 in FGD1**

P1: Especailly in this area (in the IDP center) we are not well acquainted with the society and this area is somewhat strange for us and it is remote and very frightening (scary) to move around. We might communicate well with similar language with the surrounding community but some males might follow you and is very difficult to negotiate with boys. This is so scary because we have seen many bad conditions and we do not forget what happened to us in the past while we were being displaced from our home. As a result, I do not dare to go far.

**1:35 ¶ 75 in FGD1**

P: The first time we came here, we heard that one woman was raped and from then onwards we are very afraid of that and we do not feel free even to bring water from the nearby or other places, or to buy other goods or to collect woods. Hence, you be afraid of raping every time you move. We prefer to move in groups or otherwise your father or brother has to be with you. Every time you plan to move, you need to be accompanied by males. If you are raped, many things will happen such as pregnancy, disease and other things.

**1:36 ¶ 77 in FGD1**

P: yes we do.we also move out of this compound to collect woods. But when you move to collect woods you need to have your brother or any close relative with you so that you will not be raped by other men……the situation is so scary.

**1:37 ¶ 79 in FGD1**

P:As it has been said it is very difficult to bring water or collect wood. You do not have the freedom to do that. You have to be with your brother or any close friend that can protect you.

**2:39 ¶ 66 in FGD2**

P4: The toilet is far away from home, and it is scary to go to there during the night. If going outside is unavoidable, for example when you are too sick, you might encounter some people who might attack/rape you. So we are so scared of sexual violence.

**2:40 ¶ 67 in FGD2**

P1: During the night, our families do not allow us not only to go outside but even to open the door. But, you make a reason that you want to go to the toilet. But you will still be scared of sexual violence or ant attack from animals. You might get drunken persons or any other that can attack you

**3:14 ¶ 72 in FGD 3**

P10: If your are living with your own family, you will inform to one of your sisters or may brothers to accompany you to go to the toilet to change your pad. But you cannot change the menstrual pad in the room because there people other than your family. If you do not have sister or brother, you will be forced to tell other people may be your peer to accompany you to the toilet. Because it is so scary to go alone to the toilet during the night.

**3:15 ¶ 73 in FGD 3**

P7:If menstruation comes suddenly in the night, you will tell to your sister or friend of similar age of same sex to accompany you to go to the toilet to change your menstrual pad. Otherwise, it is difficult to change your menstrual pad in the same room.

**3:16 ¶ 74 in IDI5**

When you go outside, you need your sister to accompany you as you cannot change it inside.

**4:9 ¶ 48 in FGD 4**

We girls are victims of Sexual violence, we suffer from menstruation, and we suffer from lack of sanitary materials and so many things.

**○ Challenges to MH_ Toilet is remote**

**7 Quotations:**

**1:28 ¶ 68 in FGD1**

P7: It is very difficult. The toilet is remote and when you got soaked with blood you might tell your mother or sister to accompany you and you might go far to change even if it is dark. Otherwise, it is difficult to change your modes in front of your family even your brothers especially in the presence of guests. You do not dare it even if you are not shy. It is very difficult to do so in our society and is not acceptable. I do not know…….it is difficult!

**1:32 ¶ 73 in FGD1**

P1: Especailly in this area (in the IDP center) we are not well acquainted with the society and this area is somewhat strange for us and it is remote and very frightening (scary) to move around. We might communicate well with similar language with the surrounding community but some males might follow you and is very difficult to negotiate with boys. This is so scary because we have seen many bad conditions and we do not forget what happened to us in the past while we were being displaced from our home. As a result, I do not dare to go far.

**2:35 ¶ 61 in IDI6**

When it comes during the night, you can do nothing. You better stay where you were. Either you sit or stand till it is down. You do not move outside because it is dark, scary and remote. Besides, this camp is remote and there are many hyenas. Hence, you will be at risk, if you go outside to change you pad. So you have no option.

**2:36 ¶ 62 in FGD2**

P1: when menstruation comes during the night, as there is no light here you spent the night with suffer. You cannot go to the toilet because it is dark, and the toilet is far and there also hyenas and dogs. So it is so scary to go outside during the night. So you simply worry and suffer at home. You can do nothing till it is down except praying to shorten the night. You cannot change inside in the presence of your families.

**2:37 ¶ 63 in FGD2**

P3: It is so challenging when menstruation comes during the night. You cannot go to the toilet as it is so far. You cannot change the pad inside your home because it is dark and as you try to search the pad in your home during the night everybody will be awaken and ask what happened to you. So you prefer keeping it till morning.

**2:37 ¶ 64 in FGD2**

P1**:** When menstruation happens at night, we suffer, because there is no light here [in the camp]. We cannot go to the toilet because it is too dark, and the toilet is far away. There are also hyenas and dogs wandering around during the night, so we just suffer and worry. We can’t do anything until it is dawn, but we pray for the night to be shorter so we can change our menstrual pads

**2:38 ¶ 65 in FGD2**

P6: Yes! As we move outside during the night, you might get hyenas or drunk person or any other man who can attack you. So, the situation is scary in here. There was no light during the night and hence, we were scared of people may be fear of sexual violence or animal attack when we go outside during the night.

**3:9 ¶ 65 in IDI3**

Let me share with you what happened to me one time. One night, as usual, there was no light in the camp, and I had to go to the toilet. I had to light a candle to get there. Unfortunately, the candle was blown out by the wind, and I got scared and had to change the modess [menstrual pad] in the dark.

**○ Community Awareness on MH**

**1 Quotations:**

**3:18 ¶ 79 in IDI1**

Oh, that (discussing menstruation) is unthinkable. Even back at home discussing menstruation is not comfortable except with my mom. When we come here, people’s concern is about safety, food, and returning home. So, we don’t discuss it (menstruation). Can’t you see how many of us are in a single tent? How can we ask for information in front of all the family members here.

**4:15 ¶ 30 in FGD 5**

We don't have information about the availability of health services …. I don’t know whether there is a health center here inside the camp. For instance, the idea of going to a clinic to get pain medication during menstruation has never come to my mind because I don’t even know if there is a clinic here (in the camp)

**○ Donation of Sanitation pads to displaced girls**

**4 Quotations:**

**2:4 ¶ 38 in IDI2**

Very rarely, some NGOs provides us menstrual pads but we do not get modes (Menstrual pads) most of the time.

**3:19 ¶ 82 in FGD 3**

P6: It would have been nice, had anybody can provide us menstrual pads and soap.

**4:1 ¶ 44 in FGD 4**

P6: while we were at home (before we were displaced), we had good practice of menstrual hygiene management. We use to get or buy menstrual pad and other sanitary materials on time. For example we used to get menstrual pads from our school while we were at home. The first time we came here, they used to give us sanitary materials to some extent. But now, we are not getting any support.

**4:4 ¶ 45 in FGD 4**

P1:While we were at home, before the war broke out, we used to get everything. When the menstruation comes, we used to manage it properly, but now we do not get pad, soap and other sanitary materials properly and on time….and we are demanding that.

**○ Girls feel disgraceful/ hate of being female**

**5 Quotations:**

**2:27 ¶ 53 in FGD2**

P6: You do not have freedom to change your modes because you cannot change your modes as many of your family members are at home. You will be ashamed of that besides the clothes we are using are so thin and cannot absorb the blood well so the blood will stain your cloth. At this time, you will be so shocked and you cannot sit with anybody else. So this is very disgraceful and you hate yourself and being female. We are so unlucky because we were created being female and we wish to be male.

**2:28 ¶ 53 – 55 in FGD2**

So this is very disgraceful and you hate yourself and being female. We are so unlucky because we were created being female and we wish to be male.

Probing: did you feel the same as you were at your own home, before you were displaced?

P6: No, we are feeling it now. Because when we were at home, we had the access to everything. But in here how can you prevent it, you no modes or anything else. The only option you have is to use clothes but the cloth is not good enough to absorb the blood. You might tear many clothes but that is useless.

**4:3 ¶ 44 in FGD 4**

When the menstruation comes, you get disturbed and bored. Because you do not have pads. This is making us to hate ourselves to the extent that you wish that you were not created.

**4:6 ¶ 46 in FGD 4**

P2: Women are being challenged by many things. After we were displaced from home, We are not getting everything we need in here. When we were moving here, we suffer a lot. Because, we did not have sanitary materials. The blood leaked in to our clothes and it is embarrassing.

**4:15 ¶ 53 in FGD 4**

P4: In this camp, males and females are living together in the same room. For the males, they do not have any problem as they do not have menstruation but we females do have menstruation. So, the only chance we get to wash our menstrual pads is during the night, but the problem is how and where can you hang it up to dry? Hence, when males and females sleep together in the same room, it is difficult. We need support, we need even psychiatrist doctor. Our mind is hurt…..in fact everybody in Tigray is hurt but we are more hurt and we females need special treatment and need separate place to live. We should not be mixed (put together with males in the same room). We should sleep in different rooms. This is what I can say.

**○ Girls feel embarrassed**

**1 Quotations:**

**3:2 ¶ 56 in FGD 3**

P: The menstrual hygiene management is quite different here and there. Even if you are out of your home, you need water, soap, and menstrual pad to keep your menstrual hygiene. If you do not have menstrual pad, your clothes will be stained with blood, you cannot participate in social affairs and you cannot meet people…and that is so embarrassing.

**○ Girls feel embarassed of**

**1 Quotations:**

**3:2 ¶ 56 in IDI3**

The menstrual hygiene management is quite different here and there. Even if you are out of your home, you need water, soap, and menstrual pad to keep your menstrual hygiene. If you do not have menstrual pad, your clothes will be stained with blood, you cannot participate in social affairs and you cannot meet people…and that is so embarrassing.

**○ Girls feel menstruation as a curse**

**3 Quotations:**

**2:18 ¶ 44 in FGD2**

P1:We do not even find the old clothes easily. While we were at home, we used to find clothes as needed but in here we have no extra clothes. We cannot tear clothes for that purpose as there is critical shortage of clothes. We suffer a lot because of menstruation and we always curse the day we were borne.

**3:21 ¶ 86 in IDI 3**

I came on foot to this camp, crossing up and down the country. While I was traveling, my period appeared, and I really cursed it because I had nothing to use to absorb it. Before this time [before war and displacement], I was eager to see it every month. However, when menstruation appeared while I was traveling [during displacement], I hated myself for being a female…oh… [Sigh of desperation].

**3:22 ¶ 87 in FGD 3**

P1: Yes, while I was travelling to here, I was so worried and I wished that would not have happen. I did have any cloth to change and it was so challenging. But before that I was eager to see it every month….I question myself why the menstruation is not coming? እንድሕር ዘይመፀ ግን ኣይመፂ ድዩ ይብል

**○ Girls feel Trouble about MH**

**13 Quotations:**

**1:2 ¶ 41 in FGD1**

But now we are using pieces of clothes for this purpose and we are in trouble.

**1:13 ¶ 45 in FGD1**

it……emm there are many problems with it.

**1:14 ¶ 45 in IDI1**

Menstruation is a blessing gifted for females. But now we are displaced from our homes and we are in difficulties. You know what I wish every time my menstruation occurs here in the camp…? I wish I had never been born, or that I had been born male! … Anyway… we have no option except to accept nature

**1:23 ¶ 59 – 60 in FGD1**

Probing: Do you get worried about as we worry about for food or anything else. For example we females have additional burden than males?

P6: yes, we worry as the menstruation period comes. You do not have the freedom and you got worried. We do not dry it outside with freedom like other clothes. As my colleagues mentioned, more modes are thrown away……

**5:20 ¶ 45 in IDI 3**

..Once, my youngest brother felt severely ill with diarrhea and vomiting, but the government health center nearby did not have the necessary medication, so we had to purchase it from a private drug seller. Unfortunately, we (the family) did not have enough money to buy the medication, but something sprang into my mind: I could sell the menstrual pads that I had received. I had to raise the amount of money we needed. It was difficult, but it was necessary to save my brother's life. I am sure I would not die from using old clothes for menstrual pads, but my brother could have died..

**1:24 ¶ 62 in FGD1**

P8: yes, I think of it so many times. I wish that every time the menstruation comes. I wished as if I was not created or otherwise created as male. Anyways, as it has been said, you pass it by using pieces of torn pyjamas or any other clothing…… Everything passes.

**1:25 ¶ 63 – 64 in FGD1**

Probing: how are you passing this time?

P8: Very difficult. There is big difference when you were at your own home and here.

**2:21 ¶ 45 in FGD2**

While I was at my home before I came here, I used to get medicines to manage my pain during menstruation. But now I have no option and I do not get the medicine. So I have to suffer the pain and sleep for three consecutive days till the menstruation ends.

**2:32 ¶ 58 in FGD2**

P2: As it has been mentioned, drying after washing the menstrual pad is embarrassing. Hence, it is very likely that you use the pad while it is wet. So we try hard to hang it inside the temporary housing for the day but the problem is you have no reserve to use. You cannot dry it outside, because you have no freedom to do so as many people can see it. So the situation is very difficult in here.

**3:1 ¶ 55 in FGD 3**

P7: regarding menstrual hygiene management, it is different what we had at home as compared to the practice in here. At our own home, we used to take care of our hygiene because we had the access to soap, menstrual pad and other things. But in here, we cannot find that and we are not taking care of ourselves.

**3:3 ¶ 57 in FGD 3**

P2: While we were at our home (before we were displaced) we used to keep our menstrual hygiene. Everything was ready….we had the access to soap, Menstrual pad, and pants. But now, we had nothing at hand. Because we threw away everything we had after the war broke out. But after we came here, we are in great sufferings. When people saw at you, you do not feel confident because of that.

**4:9 ¶ 48 in FGD 4**

We girls are victims of Sexual violence, we suffer from menstruation, and we suffer from lack of sanitary materials and so many things.

**4:14 ¶ 52 in FGD 4**

P1: we are living in this room with many people. When your menstruation comes, it is difficult to change and wash your menstrual pad. So you have to wait till all people leave the room so that either you will wash or change your menstrual pad. We are really challenged in this camp. The situation is very very difficult and I am so much worried. Because of this, these days my menstruation comes every two weeks or less, I do not know when it will comes it just comes all of a sudden. I need to see a doctor.

**4:16 ¶ 54 in FGD 4**

P3: When you sleep in the same room with your brother, or any of your neighbor or anyone who you might know, how can you change your pad or cloth if your menstruation comes suddenly during the night and your clothes might be soaked. It might come without feeling pain or without any prior sign. So you need soap, water and Pad. So it’s difficult to do such things.

**○ Girls restrict social participation b/c of manustration**

**1 Quotations:**

**3:4 ¶ 58 in FGD 3**

P4: As it has been said, while we were at home we used to wash the washable menstrual pads regularly and timely. But in here, there is no soap and you cannot wash your menstrual pad and your clothes will be soaked and this affects your social relationships.

**○ Girls do nothing for MH**

**2 Quotations:**

**3:17 ¶ 76 in FGD 3**

P:Yes, there is. Previously, immediately of the war broke out, we did not get the chance to use pad or any other cloth. We were simply bleeding. But, it is a little bit better now than then.

**3:20 ¶ 85 in FGD 3**

P6: when you bleed, especially if you have not pad, you wish so. Especially when were were displacing and travelling by car to here, I saw many women bleeding. So many of them were embarrassed and many of them were torched psychologically. So they did not clothes to change, a water to wash and it was so difficult.

**○ MH practice among Displaced Girls**

**0 Quotations**

**○ People's reaction to menstruation**

**1 Quotations:**

**4:8 ¶ 48 in FGD 4**

As a result, our clothes become soaked with blood suddenly and the people do not understand our problem and are pointing their finger at us when they see blood stained clothes on us. They blame us as we are dirty and lazy to keep our menstrual hygiene. They try to embarrass us in front of people. Hence, the government should understand it. This war has enormous impact on us. On top of everything, we girls are suffering a lot.

**○ What do displaced girls use for MH_ Cloths**

**7 Quotations:**

**1:1 ¶ 41 in FGD1**

Before now, before the war broke out, we were using the non-washable or normal modes most of the time. But now we are using pieces of clothes for this purpose and we are in trouble.

**2:1 ¶ 37 in FGD2**

P6:emm…when menstruation happens, while we were at our home (before we were displaced), we used to use modes but now we cannot get modes and we are using pieces of clothes.

**2:3 ¶ 38 in FGD2**

P1: Before now (when we were at our home), we used to buy modes during menstruation. But now we are using pieces of clothes.

**2:5 ¶ 38 in FGD2**

We use pieces of clothes and you will be ashamed of using that because you do not get water and soap,

**2:11 ¶ 40 in FGD2**

regularly during menstruation and we used to get menstrual pads easily whenever you want. But now we are using the washable pads and we are not getting soap…..and

**2:14 ¶ 41 in FGD2**

We even try to get soft clothes instead but that is also difficult to find and use. Anyways having menstruation in such kind of environment is very challenging.

**2:16 ¶ 42 in FGD2**

Therefore, we are forced to use pieces of clothes
